# Micro-deformation evolutions of the constituent phases in duplex stainless steel during cyclic nanoindentation

**DOI:** 10.1038/s41598-018-24589-4

**Published:** 2018-04-18

**Authors:** Yuan-Yuan Cui, Yun-Fei Jia, Fu-Zhen Xuan

**Affiliations:** Key Laboratory of Pressure System and Safety, MOE, School of Mechanical and Power Engineering, East China University of Science and Technology, Shanghai, 200237 P.R. China

## Abstract

Cyclic elastoplastic deformation behaviors of austenite phase and ferrite phase in a duplex stainless steel were investigate by load-controlled cyclic nanoindentation with a Berkovich indenter. During the tests, the maximum penetration depth per cycle increased rapidly with cycle number at transient state, and reached stable at quasi-steady state. Plastic dissipated energy was quantitatively proved to be the driving force for the propagation of deformation zones during cyclic nanoindentation tests. Transmission electron microscopy combined with FIB was used to reveal the deformation mechanisms of both phases underneath indents with cycles. After quasi-static single loading, nucleation and concentration of dislocations were observed in both austenite phase and ferrite phase under the indenter. After cyclic loading, dislocations propagated to further regions in both phases. Besides, slip bands were generated within single nanoindentation and propagated during the subsequent cyclic nanoindentation. The sizes of the deformation regions for both phases under the indents after cyclic indentation observed by TEM were consistent with those calculated by the expansion model of spherical cavity.

## Introduction

Duplex stainless steels (DSS) are composed of approximately equal amounts of two constituents, such as austenite phase and ferrite phase, or ferrite phase and martensite phase. Due to their duplex phase microstructures, DSS combine the beneficial properties of their constituents. For instance, DSS exhibit high strength and great fatigue property in corrosive environments^1–3^. Their excellent properties make them widely used in petro-chemical, transportation and other industries. In this work, we limit the discussion to duplex steels composed of austenite and ferrite as they constitute the most relevant microstructure.

To have a deeper understanding of the mechanical properties of DSS, it is necessary to investigate the local deformation behaviors of each constituent phase at the scale of crystalline. However, it’s difficult to use the conventional mechanical testing techniques, such as uniaxial tension-compression^4^, bend^5^ and torsion^6^ tests, to measure the mechanical properties of the constituent phase in a duplex stainless steel. Due to the simple operation, nanoindentation test has been widely used to characterize the local mechanical properties, such as elastic modulus and hardness^7–11^, and local deformation behaviors of materials at micro/nano-scale, such as the load-displacement curve^12^, the viscous-elastic-plastic behavior^13^. By the nanoindentation tests performed onto the constituent phases in austenitic-ferritic duplex stainless steels, the elastic modulus and hardness of ferrite phase are greater than those of austenite phase^14,15^. In order to figure out the deformation mechanisms of materials under the indentation pit, some researchers have used microscopy observation, such as Scanning Probe Microscope (SPM), Scanning Electron Microscope (SEM) and Transmission Electron Microscope (TEM), combined with sample preparation technology of Focused Ion Beam (FIB) milling. Chen *et al*.^16^ studied the pile-up profile around the indentation sites with different loading rate, and explained this phenomenon with the dislocation theory. Xie *et al*.^17,18^ performed *in situ* nanocompression of α-Fe nanopillars, and observed the movement/escape of dislocations and the formation of slip bands, which induced a series of small- and intermediate- scale strain bursts. Misra *et al*.^19^ probed the deformation behaviors under the impressions of 316L, 301LN, and TWIP austenite steels, in which the nucleation of dislocations in all three austenite steels, activities of dislocations and twinning in stable 316L stainless steel, strain-induced martensite transformation in metastable 301LN steel, and twinning in TWIP steel were observed.

As reviewed above, most studies have been focused on the quasi-static contact deformation behaviors of applied materials. However, due to the external cyclic load in service and the loading-unloading processes in the starting-up and closing-down, the device and components have to experience the dynamic behaviors in use. It is necessary to investigate the cyclic deformation behaviors of the materials at the micro/nano-scales. The numerical and experimental studies of cyclic indentation have been used to investigate the contact fatigue behaviors of materials. Yang *et al*.^20^ used the cyclic indentation tests to observe the dynamic deformation of aluminum, and they observed that the penetration depth experiences the transient state and the quasi-steady state. Jia *et al*.^13^ performed the cyclic indentations to investigate the dynamic behavior of the tooth enamel, and they suggested that the amplitude of the indentation depth and the penetration depth per cycle for the cyclic indentation of the axial section were larger than those for the indentation of the occlusal section under the same loading condition. Amini *et al*.^21^ suggested that the decrease of hysteresis energy and dissipated energy could characterize the propagation velocity of interface, thermal activation volume and the levels of phase transition stress for the cyclic indentation on nanocrystalline NiTi Shape Memory Alloy. Jia *et al*.^22^ simulated the cyclic indentation of the tooth enamel, and found the inner enamel provided better crack/fracture resistance due to its larger equivalent plastic strain and lower stresses in comparison with the outer enamel. Nevertheless, to our knowledge, few studies focused on the cyclic indentation behaviors of the constitute phase of duplex stainless steel and on the deformation mechanisms evolution underneath the indent with indentation cycles. In this work, the load-controlled cyclic indentation tests on the respective phase of an austenite/ferrite duplex stainless steel were performed to investigate the dynamic deformation behaviors of austenite phase and ferrite phase, and TEM observations combined with FIB milling technology were used to reveal the deformation mechanisms of the respective phases under indentations with cycles. The differences in the evolutions of mechanical properties and mechanisms with cycles between the two phases are the focus in the present study. The present study offers a better understanding of cyclic deformation behaviors and mechanisms of austenite phase and ferrite phase at the micro scale.

## Methods

### Material and sample preparation

The chemical compositions of constituent phase of the as-received commercial duplex stainless steel from Baosteel Stainless Steel Co., Ltd. were shown in Table S1. The specimen for the indentation was firstly machined by wire-cut to get a cuboid with the size of 20 mm × 20 mm × 8 mm, and then ground with SiC papers from 400 to 2000 grit to remove the mechanically damaged layer. Then, the surface was mechanically polished with 0.06 μm Colloidal Silica Suspension until no observable scratches, and finally electrolytically etched in 20% sodium hydroxide solution to clearly distinguish austenite phase and ferrite phase under the optical microscope. The surface roughness of tested region was ensured below 100 nm to make sure that the effects on the penetration depths can be neglected. Three-dimensional microstructure of studied duplex stainless steel exhibits that the austenite grains with the size in the range of 8–25 μm are embedded in the continuous ferrite phases, as shown in Fig. S1(a). Determined from manual point counting in accordance with the ASTM E562-11 standard^23^, the volume percentages of austenite phase and ferrite phase are about 58% and 42%, respectively.

### Cyclic indentation tests and survey scanning

Cyclic indentation tests were carried out by Agilent Nano Indenter G200 (Agilent Technologies Inc., Santa Clara) at room temperature, imposed by the Berkovich indenter with the tip radius less than 40 nm. Cyclic indentation tests were conducted under load controlled mode at the same loading/unloading rate, $\dot{P}=dP/dt$, of 40 mN/s. The maximum indentation load per cycle, ${P}_{max}^{i}$, which has to be large enough to eliminate the size effect and the influence of grain orientation, was defined as 280 mN. Yang *et al*.^20^ pointed out that, for small amplitude of the indentation load, shakedown phenomenon of the penetration depth might occur and the cyclic indentation was primarily controlled by the elastic deformation. The plastic deformation, such as dislocation multiplication and propagation, being activated at the edge of the contact zone during the repeated loading, could be regarded as secondary. Hence, the amplitude of the indentation load per cycle, Δ*P*, was defined as 150 mN. The loading/unloading path was a triangular wave as shown in Fig. S1(b). According to the study of Jia *et al*.^13^, the penetration depth existed two states of transient state and quasi-steady state. To ensure that the penetration depth reached quasi-steady state, the loading/unloading cycle was set as 300 times. Extra cyclic indentation tests were performed to investigate the effects of the maximum indentation load and amplitude of the indentation load on cyclic deformation behaviors of the austenite phase and ferrite phase. The loading conditions were presented in Table S2.

To diminish the influence of adjacent phase and grain boundaries on the experimental results, all indentions performed in this study were located at the center of individual phase. After the cyclic indentation tests, the morphology of indents was characterized by the Survey Scanning technology, similar to SPM, of Agilent Nano Indenter G200 with scanning load of 8 × 10^−3^ mN (less than 0.003% of *P*_max_). Due to the anisotropic characteristic of stainless steel, 12 indents in each phase, which contribute in different grains, were conducted for each loading condition. All the trend analysis of the experimental data is the result of statistical average, without considering the influence of grain orientation.

### FIB milling and TEM observation

To investigate the deformation mechanisms evolution of austenite phase and ferrite phase during the cyclic indentation tests, the cross sectional TEM thin foils beneath the indents were milled by a dual-beam Focused Ion Beam with Scanning Electron Microscope (ZEISS Auriga SEM/FIB Crossbeam System) technique. The process of FIB was shown in Fig. S2. The foils fabricated for both phases were located at the center of the indentation axis and directed to one of three sides of the indent to make sure that the cross section was right below the middle of the indent (as shown in Fig. S2(a) and (b)). Before the milling process, a protective platinum layer about ~1 μm thickness and ~10 μm length was deposited on the indent surface to protect the specimen surface from the ion beam damage (as shown in Fig. S2(c)). Then, a Ga^+^ beam was accelerated at 30 kV with a current of 100 pA to dig the hole on both sides of the protective platinum layer to form a primary thick wall through the middle of the indent. Subsequently, finer Ga^+^ current value of 50 pA, 20 pA, 10 pA and 10 nA at 30 kV were used to obtain a thinning foil less than 100 nm thickness. A Ga^+^ beam, which was accelerated at 5 kV with a current of 10 nA, was applied to remove the damage layer formed by the previous high energy milling processes. Finally, the thin foil was moved onto the Cu grid for TEM observation (as shown in Fig. S2(d)). The micro-deformation mechanisms of austenite phase and ferrite phase under the cyclic indentation were investigated by TEM (JEOL JEM-2100F) operated at 200 kV.

## Results and Discussion

### Deformation behaviors evolution

**Figure 1:**
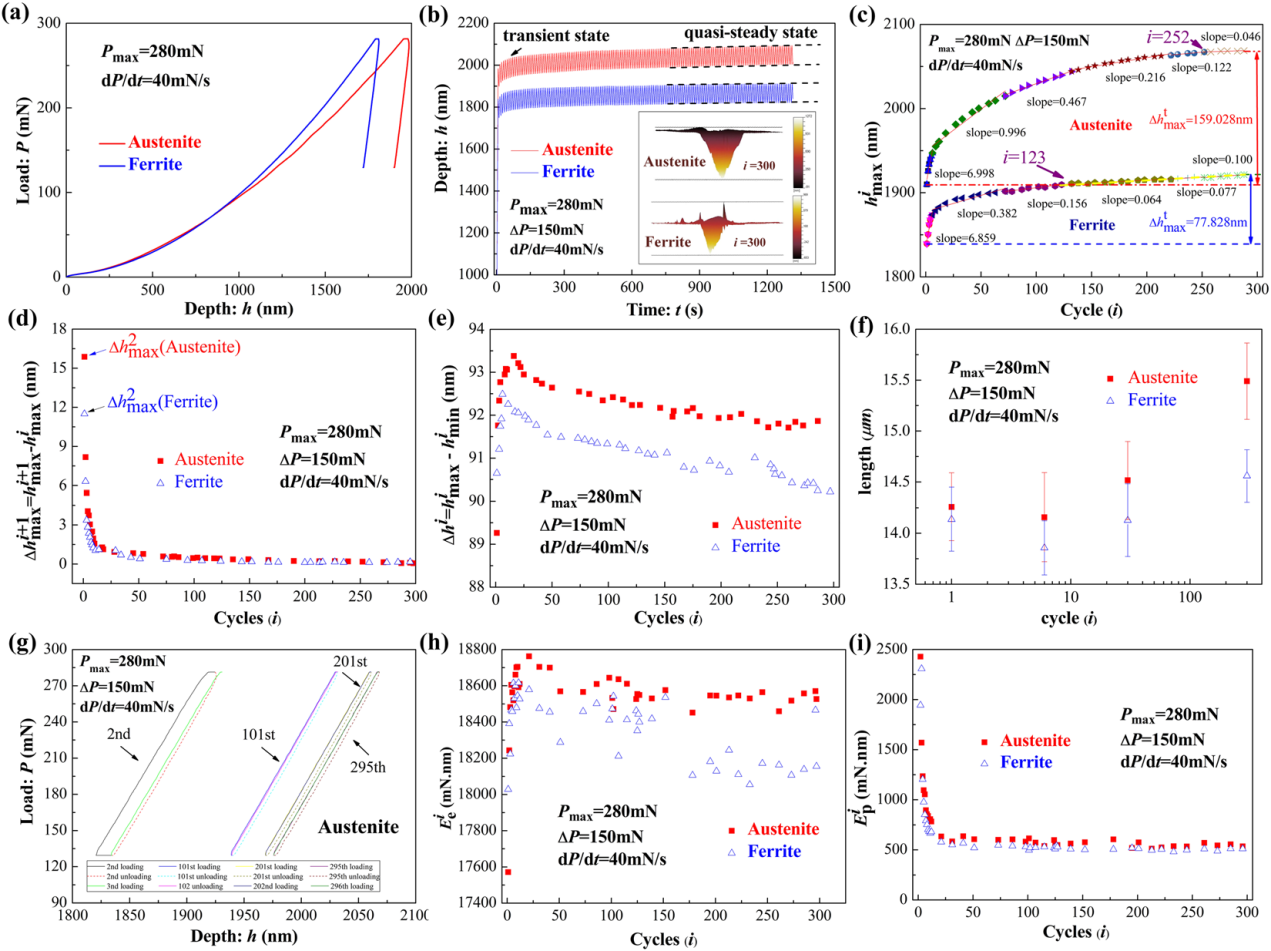
Quasi-static cyclic indentation tests on each constituent. (**a**) Indentation load vs. penetration depth curves of the 1^st^ cycle; (**b**) Penetration depth vs. time curves of cyclic indentation tests; (**c**) The maximum penetration depth per cycle vs. cycle number; (**d**) Increment of the maximum penetration depth per cycle vs. cycle number; (**e**) Amplitude of penetration depth per cycle vs. cycle number; (**f**) Length of the indents vs. cycle number; (**g**) Hysteresis loops of the 2^nd^, 101^st^, 201^st^ and 295^th^ cycle of austenite phase; (**h**) Elastic recovery energy vs. cycle number; (**i)** Plastic dissipated energy vs. cycle number.

During the quasi-static single indentation tests, the indentation load, *P*, applied on the indenter, was defined as a linear function of time, *t*, and the penetration depth, *h*, was a consequence of the indentation response of materials, which depended on the mechanical properties of tested materials and the applied loading condition, such as loading rate and temperature. Table 1 shows the elastic modulus and hardness of austenite phase and ferrite phase calculated by Oliver-Pharr method^7,8^, which are consistent with the results in the literatures^14,15^. The elastic modulus and hardness of ferrite phase are greater than those of austenite phase. Figure 1(a) shows the representative indentation load vs. penetration depth of the respective phases under the same single loading condition with the maximum indentation load, *P*_max_, of 280 mN and the loading rate, $\dot{P}$, of 40 mN/s. In comparison, the penetration depths of the indenter onto ferrite phase were shallower than those of the indenter onto austenite phase under the same indentation load, which implied that ferrite phase had better penetration resistance, i.e. higher hardness.

**Table 1:** Values of elastic modulus and hardness of austenite phase and ferrite phase measured from nanoindentation tests.

Parameter	Ferrite	Austenite
Elastic Modulus(GPa)	248.3 ± 8.7	225.4 ± 11.9
Hardness(GPa)	6.14 ± 0.33	5.79 ± 0.14

Figure 1(b) shows the variation of the penetration depth of the indenter onto austenite phase and ferrite phase with time during the cyclic indentation tests under the same loading condition with *P*_max_ of 280 mN and the amplitude of indentation load, Δ*P*, of 150 mN. During the load-controlled cyclic indentation tests, the penetration depth fluctuated periodically under the action of cyclic indentation load: in each cycle, the penetration depth increased to the maximum penetration depth per cycle, ${h}_{max}^{i}$, where the superscript *i* is the cycle number, with the increasing of the indentation load during the loading phase, and decreased to the minimum penetration depth per cycle, ${h}_{min}^{i}$, with the withdrawing of the indenter during the unloading phase. Similar deformation behavior was also observed in ferrite phase, nothing but the shallower maximum penetration depth in ferrite phase occurred in comparison with that in austenite phase at each cycle due to the better penetration resistance of ferrite. With the increase of the cycle number, ${h}_{max}^{i}$ for the indentation of austenite phase and ferrite phase increased continuously.

As well known, Johnson^24^ postulated that the analysis for the expansion of a spherical cavity in an elastic-plastic material could be applied to the hemispherical radial mode of the deformation observed in indentation tests by replacing the cavity with an incompressible, hemispherical core of material directly beneath the indenter of radius equal to the contact circle, *a*_c_, as shown in Fig. S3. Outside the hydrostatic pressure zone, a hemispherical plastic zone of radius, *c*, is the elastic-plastic deformation zone. For geometrically similar indentations, like a conical indenter, the radio of *c/a*_*c*_ is a constant for all values of load and penetration depth, dependent on the elastic modulus, yield strength, Poisson’s ratio of the tested material.

According to the mathematical relationship between the radius of the hydrostatic pressure zone, *a*_*c*_, and the depth of the circle of contact from the specimen free surface, *h*_*a*_, the value of *a*_*c*_ can be calculated as:

1 $${a}_{c}\approx a={h}_{a}\,\tan \,\alpha $$

where *α* is the angle of inclination of the indenter with the specimen surface. Here, the value of *h*_*a*_ can be calculated as half of the maximum penetration depth, *h*_max_:

2 $${h}_{a}=\frac{1}{2}{h}_{max}$$

Hence, for the triangular pyramid Berkovich indenter in this study, the values of *a*_c_ are about 1.13 μm and 1.05 μm for austenite phase and ferrite phase, respectively, under the single loading condition of *P*_max_ = 280 mN. Moreover, the values of *a*_c_ are about 3.18 μm and 2.96 μm for austenite phase and ferrite phase, respectively, under the cyclic loading condition of *P*_max_ = 280 mN, Δ*P* = 150 mN and *i* = 300.

According to the study of Hill^25^, the ratio of the plastic zone and the hydrostatic pressure zone for the elastic-plastic materials can be calculated as:

3 $$\frac{c}{a}={[\frac{4(1-2\nu )+(E/Y)tan\alpha }{6(1-\nu )}]}^{1/3}$$

where *E*, *Y*, *ν* are the elastic modulus, yield strength, poison’s radio of the tested material. The radio of *c*/*a* are calculated to be 9.01 and 5.93 for austenite phase and ferrite phase, independent of loading condition. Hence, for austenite phase, the values of *c* are about 10.19 μm and 28.65 μm after 1 cycle and 300 cycles, respectively. For the ferrite phase, the values of *c* are about 5.93 μm and 17.55 μm after 1 cycle and 300 cycles, respectively.

Figure 1(c) shows the variation of the maximum penetration depth per cycle, extracted from *h* vs. *t* curves shown in Fig. 1(b), with the cycle number during cyclic indentation tests on austenite phase and ferrite phase under the action of indentation load of *P*_max_ = 280 mN and Δ*P* = 150 mN. The value of ${h}_{max}^{i}$ increased significantly with the cycle number at the first few cycles, i.e. experiencing the transient state, and then increased steadily after a certain cycle number, i.e. reaching the quasi-steady state. Obtained from the equations (1) and (3), the distribution depth of the hydrostatic pressure zone and the plastic deformation zone increased synchronously with the increase of ${h}_{max}^{i}$.

The red lines are the best linear fitting for every stage. As clearly demonstrated in the Fig. 1(c), the increasing rate of ${h}_{max}^{i}$ for the indentation of both austenite phase and ferrite phase decreased with the cycle number. The increasing rate of ${h}_{max}^{i}$ of ferrite phase first decreased rapidly with the cycles and then approximately approached constant, while the increasing rate of ${h}_{max}^{i}$ for the indentation of austenite phase gradually decreased to be stable. Ferrite phase reached the quasi-steady state faster than austenite phase (about 123^rd^ cycle and 251^st^ cycle, respectively). Moreover, the total amplitude of the maximum penetration depth, ${\rm{\Delta }}{h}_{max}^{t}={h}_{max}^{300}-{h}_{max}^{2}$, for the indentation of austenite phase, inserted in Fig. 1(c), was twice as much as that for the indentation of ferrite phase. The variation of increasing value of the maximum penetration depth per cycle, ${\rm{\Delta }}{h}_{max}^{i+1}={h}_{max}^{i+1}-{h}_{max}^{i}$, is shown in Fig. 1(d). The maximum value of ${\rm{\Delta }}{h}_{max}^{i+1}$ for the indentation on both austenite phase and ferrite phase occurred at the 2nd cycle, i.e. ${\rm{\Delta }}{h}_{max}^{2}$. The value of ${\rm{\Delta }}{h}_{max}^{i+1}$ decreased rapidly with the cycle number at the transient state, and then decreased slowly at the followed quasi-steady state. The difference between the increasing value of the maximum penetration depth per cycle of austenite phase and the value of ferrite phase was not obvious, but still nonnegligible. The values of ${\rm{\Delta }}{h}_{max}^{t}$, ${\rm{\Delta }}{h}_{max}^{2}$, and ${\rm{\Delta }}{h}_{max}^{s}$ for the indentation of austenite phase and ferrite phase are shown in Table 2. All the values for the indentation of ferrite phase are less than those of austenite phase, suggesting that the ferrite phase has a greater cyclic penetration resistance.

**Table 2:** The total increasing value of ${h}_{max}^{i}$, ${\rm{\Delta }}{h}_{max}^{t}$, the maximum value of increasing value of ${h}_{max}^{i}$, ${\rm{\Delta }}{h}_{max}^{2}$, and the steady value of increasing value of ${h}_{max}^{i}$, ${\rm{\Delta }}{h}_{max}^{s}$, for the cyclic indentation tests on austenite phase and ferrite phase.

	Ferrite	Austenite
${\rm{\Delta }}{h}_{max}^{t}$(nm)	77.828	159.028
${\rm{\Delta }}{h}_{max}^{2}$(nm)	11.502	15.879
${\rm{\Delta }}{h}_{max}^{s}$(nm)	0.139	0.172

As mentioned above, during the unloading phase, with the decrease of the indentation load, materials under the indenter experienced the elastic recovery. The elastic recovery per cycle was quantitatively expressed by the amplitude of the penetration depth, ${\rm{\Delta }}{h}^{i}={h}_{max}^{i}-{h}_{min}^{i}$. The variation of ${\rm{\Delta }}{h}^{i}$ with *i* for the indentation of austenite phase and ferrite phase under the same cyclic indentation load is shown in Fig. 1(e). ${\rm{\Delta }}{h}^{i}$ for the indenter onto austenite phase and ferrite phase experienced increasing stage before decreasing with the cycle number, which meant the elastic recovery increased before a certain cycle number, and then decreased with the cycle number. From Fig. 1(e), one can find out that the certain cycle number of austenite phase and ferrite phase were ~16 and ~6, respectively. Besides, ${\rm{\Delta }}{h}^{i}$ of ferrite phase could reach the quasi-steady state faster than that of austenite phase under the same cyclic loading condition.

Learning from the equations (1) and (3), the distributions of the deformation zones under the indenter, contained hydrostatic pressure zone, plastic deformation zone and elastic deformation zone, are only proportional to the penetration depth. Specifically, the deformation zones continually propagate to the deep region with the increase of the penetration depth during the loading process, but recover when the penetration depth decreases during the unloading process. The propagation-recovery process of deformation zones under the indenter could be reflected by the scale of impression during cyclic nanoindentation. Figure 1(f) shows the variation of the length of the residual indents, *L*, with the nanoindentation cycles on austenite phase and ferrite phase, respectively. The tendency of the length of the residual indents after different cycle number are completely contrary to the trend of the recovered amplitude of the penetration depth shown in Fig. 1(e) and the trend of the elastic energy shown in Fig. 1(h).

During the cyclic indentation tests on the respective phase, the recovered amplitude of the penetration depth and the elastic recovered energy per cycle increase before the ~16^th^ cycles for austenite phase and the ~6^th^ cycles for ferrite phase, and then decrease with the cycle number. The indents on both phases at the end of the 6 cycles have the shortest lengths of all indents at the end of different cycles, which manifest that larger value of the elastic recovery leads to more recovery of deformation zones underneath the indenter and leaves a smaller impression. Then, after 6 cycles, the lengths of the indents on the respective phase increase with the cycle numbers. As shown in Fig. 1(c) and (e), with the increase of the cycle number after a certain number, the maximum penetration depth per cycle increases, but the elastic value of the penetration depth per cycle decreases, which causes that the deformation zone propagates to the deeper region during the loading progress but recovers less during the unloading process. This explains why the impressions grow with the cycle number after the 6 cycles.

The lengths of the indents on austenite phase and ferrite phase have the same evolution with the cycle number. However, the experiment results of nanoindentation tests, listed in Table 1, show that ferrite phase has larger elastic modulus and hardness than austenite phase, which results in better penetration resistance of ferrite phase. Hence, under the action of same quasi-static single/cyclic indentation load, the penetration depth per cycle of ferrite phase is shallower than that of austenite phase, which causes that ferrite phase has shallower deformation zone underneath the indenter and smaller indents than austenite phase. As a result, the lengths of the indents on ferrite phase are less than those on austenite phase after the action of single load or cyclic load with same cycle number.

Figure 1(g) shows the 2^nd^ loading/unloading *P* - *h* curve, the 3^rd^ reloading *P* - *h* curve, the 101^st^ loading/unloading *P* - *h* curves, the 102^nd^ reloading *P* - *h* curve, the 201^st^ loading/unloading *P* - *h* curves, the 202^nd^ reloading *P* - *h* curve, the 295^th^ loading/unloading *P* - *h* curves and the 296^th^ reloading *P* - *h* curve of the cyclic indentation on austenite phase under the cyclic loading condition of *P*_*max*_ = 280 mN and Δ*P* = 150 mN. One can note that the unloading curves do not overlap the loading curves. In other words, there exists the hysteresis loops between the loading curves and the unloading curves, suggesting the occurrence of the dissipation of energy due to the plastic deformation under the indenter. Note that the area of closed-loop of the 2^nd^ cycle is significantly greater than that of the 101^st^ cycle. After the 201^st^ cycle, the hysteresis loop of the loading/unloading curves reaches stable. In respect of the total energy during the cyclic indentation onto the elastic-plastic materials, a considerable portion of the input energy is transferred to be the elastic energy released during the unloading phase, and the other portion is inevitably dissipated to be the plastic energy to the propagate the plastic deformation zone underneath the indentation during the cyclic indentation tests. Thus, the total energy for the *i*^th^ cycle, ${E}_{total}^{i}$, absorbed during the loading phase can be expressed as:

$${E}_{{\rm{total}}}^{i}={E}_{{\rm{loading}}}^{i}={\int }_{{\rm{loading}}}^{i}Pdh$$

which is the area under the loading curve each cycle. The elastic recovery energy for the *i*^th^ cycle, ${E}_{{\rm{elastic}}}^{i}$, can be calculated as:

$${E}_{{\rm{elastic}}}^{i}={E}_{{\rm{unloading}}}^{i}={\int }_{{\rm{unloading}}}^{i}Pdh$$

which is the area under the unloading curve in each cycle. Hence, the plastic energy dissipation for the *i*^th^ cycle, ${E}_{{\rm{plastic}}}^{i}$, can be calculated as:

$${E}_{{\rm{plastic}}}^{i}={E}_{{\rm{total}}}^{i}-{E}_{{\rm{elastic}}}^{i}=|{\int }_{{\rm{loading}}}^{i}Pdh|-|{\int }_{{\rm{unloading}}}^{i}Pdh|$$

which is the area between the loading curve and the unloading curve each cycle.

Figure 1(h) and (i) depict the development of the elastic recovery energy and the plastic dissipated energy with the cycle number of the cyclic indentation onto austenite phase and ferrite phase under the loading condition of *P*_max_ = 280 mN and Δ*P* = 150 mN, respectively. One can figure out that the released elastic energy is far larger than the dissipated plastic energy for the indentation onto both austenite phase and ferrite phase. With the cycle number increasing, the elastic recovery energy for both austenite and ferrite phase increases at the first few cycles, and then decrease to be approximately constant at the quasi-steady state. The trend of elastic recovery energy is similar to that of the amplitude of the penetration depth with the cycle number shown in Fig. 1(e). The plastic energy dissipation drastically decreases from about 2500 mN · nm to about 500 mN · nm at the few cycles and then approximately keep steady at the quasi-steady state. The trend of plastic energy dissipation is consistent with that of the increment value of the maximum penetration depth per cycle with the cycle number shown in Fig. 1(d). Both the elastic recovery energy and the plastic energy dissipation of the indenter onto austenite phase are larger than those onto ferrite phase, which is due to the lower elastic modulus and hardness of austenite phase. The values of elastic recovery energy of both austenite phase and ferrite phase are dozens of those of plastic dissipated energy, which implies that the deformation behavior is dominated by the elastic deformation for the cyclic indentation tests on both austenite phase and ferrite phase. This explained the observation that the impression became smaller at the few cycles although the maximum penetration depth per cycle increased.

Besides, the effects of the maximum indentation load and amplitude of the indentation load on the cyclic indentation behavior of austenite phase and ferrite phase were presented in Supplementary Information. At the quasi-steady state, the increasing values of the maximum penetration depth were independent of the maximum indentation load while the plastic dissipated energy decreased, but both of them increased with increasing the amplitude of the indentation load. Detailed tendency was described in from Figs S4 to S12.

### Deformation mechanisms evolution

The SEM images and the cross sections of the indents on austenite phase and ferrite phase after 300 cycles were presented in Figs S2 and 1(b), respectively. No cracking around the impressions was observed, suggesting no fracture damage was accumulated, and the pile-up phenomena exist in both phases. The physical explanation of this is that, when the materials were work-hardened during the indentation tests, the dislocation mobility was reduced and the dislocations were confined to the surface causing pile-up. The evolution of deformation behaviors underneath the indent with cycles was further investigated by TEM.

**Figure 2:**
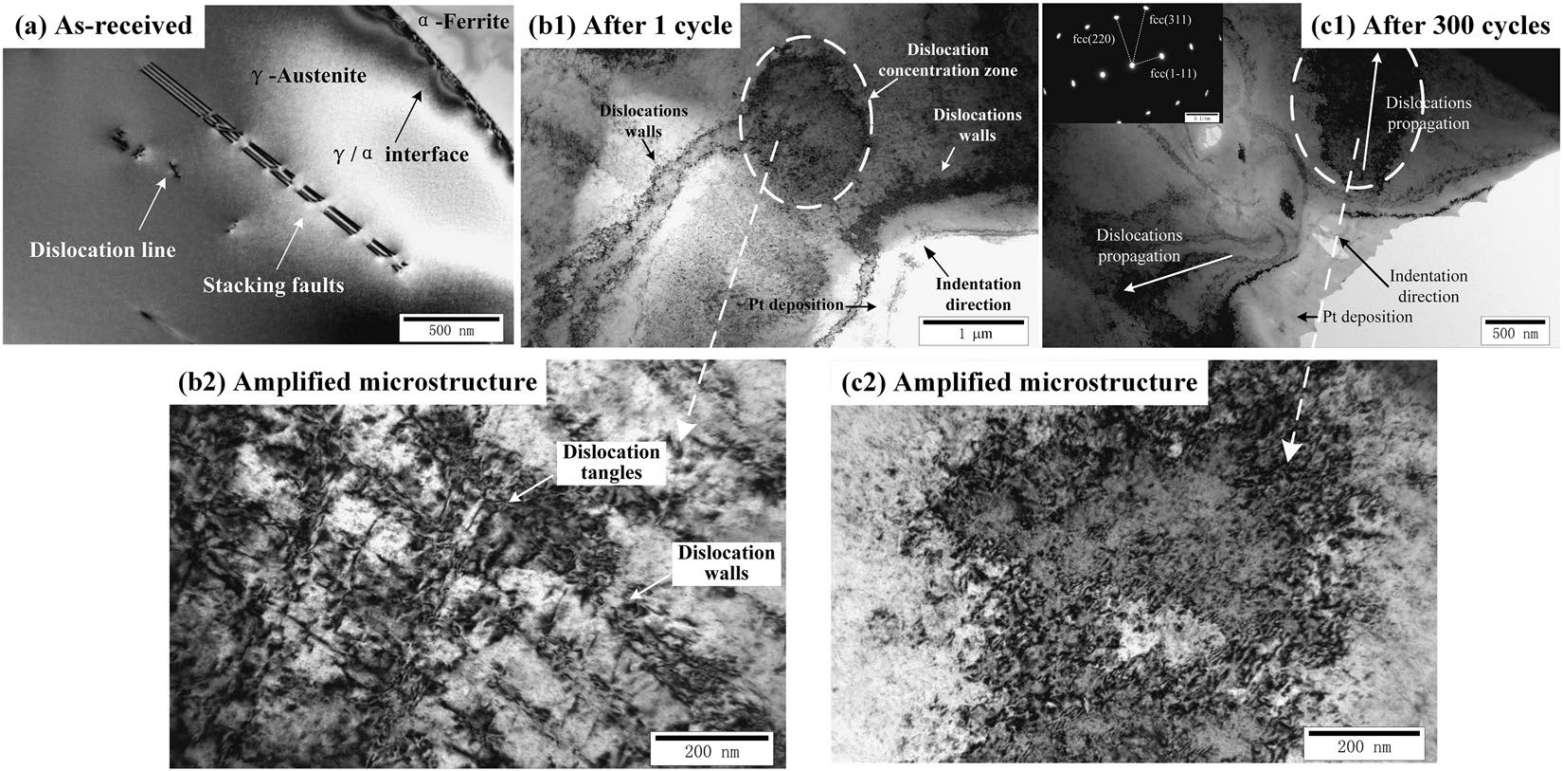
TEM images of austenite grains under different cycles. (**a**) as-received, (**b1**) and (**b2**) after 1 cycle, (**c1)** and (**c2**) after 300 cycles.

Figure 2(a–c) present TEM images of austenite grain in the as-received state, after 1 cycle and after 300 cycles, respectively. The initial microstructure of the as-received austenite grain shows the planar dislocation structures, such as discrete dislocation lines and stacking faults, as shown in Fig. 2(a). After the action of the quasi-static single load, the density of dislocations increases significantly in the stress concentration zone underneath the indenter, as shown in Fig. 2(b1), which indicates that dislocation multiplication plays an important role in the plastic deformation during the quasi-static indentation tests. The amplified microstructure of dislocation concentration zone underneath the indenter, presented in Fig. 2(b2), shows that the dislocation walls are formed and dislocation lines are tangled in austenite grains after the quasi-steady single load. To study the evolution of the deformation mechanisms of austenite phase with cyclic nanoindentation, TEM result of the austenite grain underneath the indent after 300 cycles is depicted in Fig. 2(c1). One can note that there is no significant dislocation concentration in the stress concentration zone right under the indent, and the dislocation quantities increase with the distance away from the indenter tip. The deformation behaviors suggest that specific slip planes are activated and dislocations are generated during quasi-static loading. The propagation of dislocations are the main deformation mechanisms during the action of cyclic indentation load with constant amplitude. The findings could explain why the increasing value of the maximum penetration depth per cycle (see Fig. 1(d)) and the dissipation of plastic energy (see Fig. 1(i)) are much great at the first few cycles while those are much low at the later cycles. The detailed microstructure of dislocation concentration zone after 300 cycles presents discrete dislocation lines, as shown in Fig. 2(c2). From the selected area diffraction pattern of the sub-surface under the indenter after 300 cycles, only austenite phase is verified. There was no strain-induced austenite-to-martensite phase transformation occurring in austenite grains during the cyclic indentation tests.

There are two reasons that could interpret the TEM observation results: one is the SFE level of studied material. According to the studies in the literatures^9,19,26–31^, the deformation mechanisms of austenite phase is dependent on the austenite stability. Generally, the austenite stability could be expressed by the parameter of stacking fault energy (SFE), which is related with not only chemical composition but also the temperature^32,33^. As well known, larger value of SFE means higher austenite stability. At low SFE levels (<20 mJ/m^2^) and intermediate SFE levels (20–50 mJ/m^2^), strain or stress induced martensite transformation (SIMT) and mechanical twinning (MT) are favorite deformation mechanisms in austenite steels. At high SFE levels (>50 mJ/m^2^), SIMT and MT are suppressed and the dislocation evolution is the major deformation modes^19,33,34^. Dai *et al*.^35^ expressed the relationship between SFE value and the alloy element at room temperature as:

$$\begin{array}{rcl}{V}_{SF}^{300} & = & {V}_{SF}^{0}+1.59{k}_{Ni}-1.34{k}_{Mn}\\  &  & +\,0.06{({k}_{Mn})}^{2}-1.75{k}_{Cr}+0.01{({k}_{Cr})}^{2}\\  &  & +\,15.21{k}_{Mo}-5.59{k}_{Si}-60.69{({k}_{C}+1.2{k}_{N})}^{1/2}\\  &  & +\,26.27({k}_{C}+1.2{k}_{N})\times {({k}_{Cr}+{k}_{Mn}+{k}_{Mo})}^{1/2}\\  &  & +\,0.61\times {[{k}_{Ni}\times ({k}_{Cr}+{k}_{Mn})]}^{1/2}({\rm{mJ}}/{{\rm{m}}}^{2})\end{array}$$

where *k*_*i*_ is the mass fraction of element *i*, and ${V}_{SF}^{0}$ is the virtual SFE value of pure Fe at room temperature. In the present study, the value of ${V}_{SF}^{0}$ is taken as 38 mJ/m^2^.

Based on the study of Dai *et al*. on the relationship between SFE levels and the alloy element, the SFE value of austenite phase at room temperature is about 57.76 mJ/m^2^ in this study, higher than 50 mJ/m^2^, indicating that the austenite phase studied in this work has great stability.

The other is the loading rate. It has been studied that the specimen temperature increases with the loading rate due to adiabatic heating^36^. The corresponding increase in temperature results in the increase of SFE, making the austenite more stable at high strain rates, thus suppressing TRIP. The loading rate of 50 mN/s in this study is much larger than that of 5 × 10^−3^ mN/s in these works^9,19,26–31^, which could restrain the occurrence of mechanical twinning and phase transformation during the cyclic nanoindentation. Besides, high loading rate in this study prevented the occurrence of pop-in phenomenon of the indentation load vs. the penetration depth curves during quasi-static single and cyclic indentation tests, which implied the beginning of the activation of dislocation systems, the formation of strain-induced martensite transformation and twinning^9,19^.

**Figure 3:**
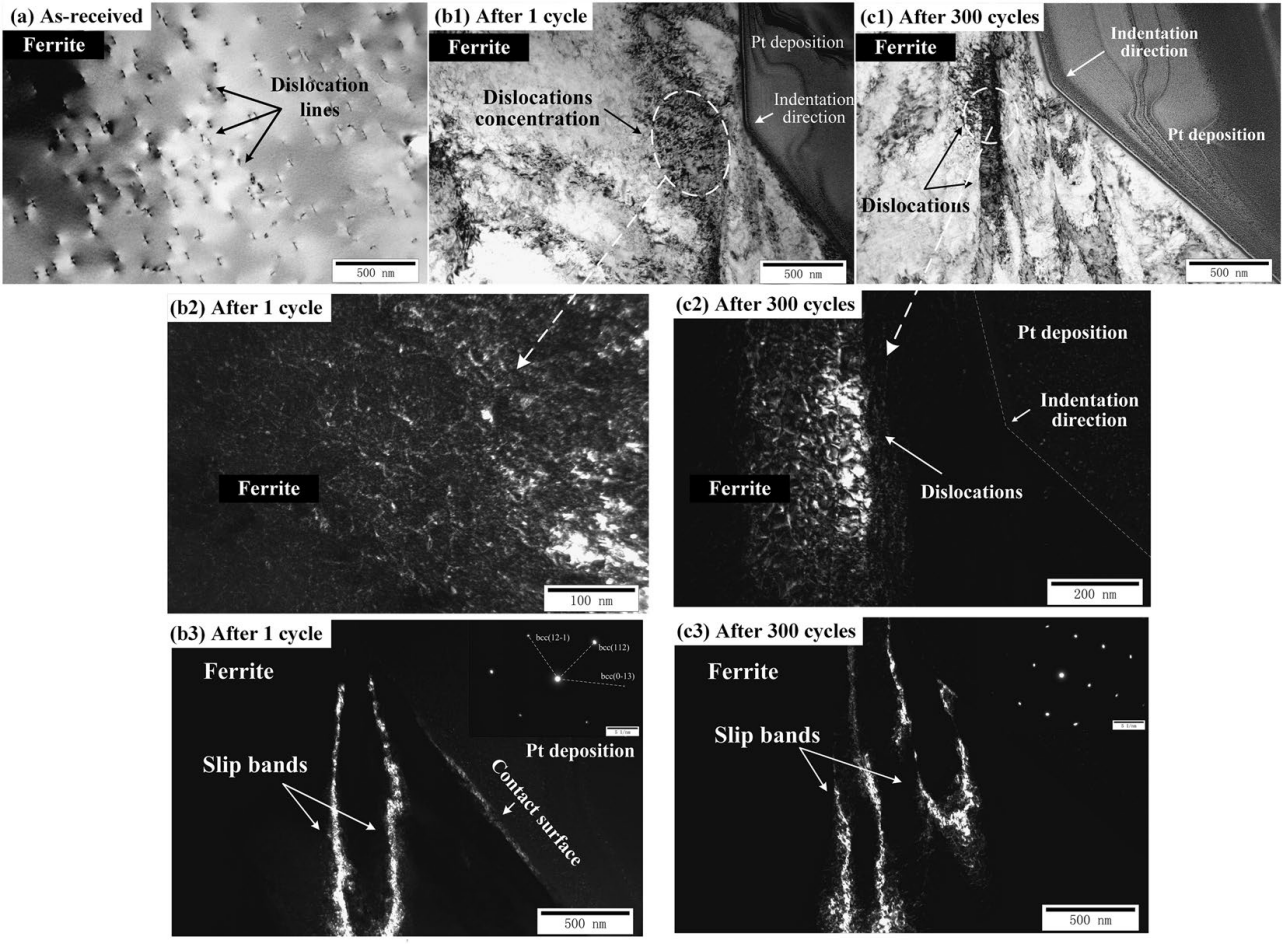
TEM images of ferrite grains under different cycles. (**a**) as-received, (**b1**), (**b2**) and (**b3**) after 1 cycle, (**c1**), (**c2**) and (**c3**) after 300 cycles.

Figure 3(a–c) present TEM images of ferrite grains in the as-received state, after 1 cycle and 300 cycles, respectively. The initial microstructure of ferrite grain before indentation contains discrete dislocation lines, as shown in Fig. 3(a). Note that the dislocation density of the as-received ferrite grain is relatively larger than that of the as-received austenite grain. Compared to the initial microstructure of ferrite grains in as-received material, the region near the indent tip shows obvious dislocation density caused by the production of dislocation on some slip planes in the stress concentration zone (see Fig. 3(b1)). Differentiating from the microstructure of austenite grains presented in Fig. 2(b1), remarkable dislocation concentration is observed in the region of ferrite grain near the indenter. This is related to better blocking ability to the dislocation propagation in ferrite phase, causing higher strength. After the action of cyclic load, the overall deformation in ferrite grains is inhomogeneous. Compared to the microstructure of ferrite grains suffered the quasi-static single load, no significant dislocation concentration is observed in the plastic deformation region near the indenter tip and dislocations grow more intensively with the increase of distance to the contact surface, as shown in Fig. 3(c1). The same deformation behavior is also observed in austenite grains after cyclic loading presented in Fig. 2(c1). Those phenomena can be explained by two speculations as followed: one is that, during the cyclic indentation process, the dislocations propagate downward several micrometers, leading to the increase of dislocation density in the deformation region far away from the contact surface; the other is the formation of slip bands in ferrite grains and the dislocation escape to the specimen surface under the cyclic loading and unloading, leading to a significant dislocation annihilation^22^. Figure 3(b2) and (c2) present the local amplified deformation microstructure of dislocations in the regions under the indent after the action of quasi-static load and cyclic load, respectively. Unlike tangling in austenite grains, after the action of quasi-static load, the dislocations distribute discretely and no dislocation wall is formed in ferrite grains. After the action of cyclic load, plenty of dislocations accumulate and numerous slip systems are active to forming several slip bands (SBs)^17^. Figure 3(b3) and (c3) present TEM dark-field images taken from deformed ferrite grains after the action of quasi-static single loading and cyclic loading, respectively, where the nucleation and propagation of SBs are observed distinctly. Those bands are possibly nucleated by the slip of the free surface. Afterwards, they propagated downwards in ferrite grains during cyclic indentation. It is noteworthy that the SBs in ferrite grains are relatively straight near the indentation surface after 1 cycle while the SBs become wavy and even tangled after 300 cycles. Moreover, more SBs generate and propagate during the cyclic indentation. The inserted selected area diffraction patterns at the top right of images suggest no twinning and phase transformation occurs in ferrite grains during both single and cyclic indentation tests. Thus, the plastic deformation of ferrite grains during cyclic nanoindentation is mainly controlled by the multiplication and movement of dislocations, as well as the collective motion of dislocations to form the SBs.

In comparison with the deformation mechanisms of austenite phase, ferrite phase exhibited higher stability, without the occurrence of SIMT and the formation of MT. According to the TEM results, the deformation mechanisms for the ferrite phase during the cyclic nanoindentation contain: 1) nucleation and propagation of dislocations; 2) formation and propagation of SBs. The microstructure of ferrite phase in the as-received materials has discrete and uniform dislocation lines. After single indentation, dislocations concentrate under the indent, indicating that the deformation process of ferrite phase is inhomogeneous. The deformation starts as elastic under loading at the first stage, then the plastic deformation occurs from the contact surface as the activation and propagation of slip systems with the increase of the indentation load. Hundreds of dislocations are nucleated and multiplicated to form the SBs, according to the observation shown in Fig. 3(b3) and (c). Fisher *et al*.^37^ proposed that the formation of SBs required ~300 dislocation loops in the single crystal, which were generated at the contact surface and terminated by their own stress field. In the present study, the magnitude of dislocations generated during the indentation loading is satisfied for the formation of SB. One can note that the SBs propagate along relatively straight lines first, and then along wavy curves. This is because hundreds of dislocations are activated and propagated along the specified directions at the first stage of loading. With the increase of the indentation load, more slip systems are activated and the generation and propagation of dislocations become chaotic in the form of wavy SBs causing wavy. During the action of cyclic indentation load, dislocations are activated at slip systems but propagate very quickly (at the speed of sound), enhancing the difficulty for the concentration of dislocations at the stress concentration zone and resulting in the formation of more SBs. Multiple slip planes are activated and the SBs become wavy and tangled at deep inside the ferrite grain.

After single indentation tests, in austenite grains (see Fig. 2(b)) and ferrite grains (see Fig. 3(b)), heterogeneous dislocations are concentrated under the indenter. After 300 cycles, dislocation concentration is absent from the deformation zone under the indenter. Differentiating from propagating of dislocations to the further region in austenite grains (see Fig. 2(c)), the discrete dislocations in ferrite phase bundle and form the SBs with a relatively higher dislocation density (see Fig. 3(c)). The SBs bands in ferrite phase after both 1 cycle and 300 cycles indicate that a considerable number of slip systems are activated. The generation of dislocations in the deformation zone under the indenter is assumed to be mainly caused by the plastic dissipated energy at the 1^st^ cycle in both austenite phase and ferrite phase. Under the action of cyclic load with constant amplitude, the deformation zones propagate to the further region as the maximum penetration depth per cycle increasing with the cycle number. The evolution of dislocations, such as dislocation nucleation and propagation, becomes the dominant deformation mechanisms under the cyclic indentation tests. Under the action of cyclic load with constant amplitude, more and more slip systems are activated with the cycle numbers. This might explain why the plastic energy in the transient state is several times the value of the quasi-steady state.

Based on the TEM observation above, during the quasi-static single and cyclic indentation tests, the major deformation mechanisms are the nucleation, multiplication and propagation of dislocations in both austenite phase and ferrite phase and the activation and propagation of slip bands in ferrite phase. In comparison with the TEM results, one can note that the deformation behaviors of austenite phase and ferrite phase during the quasi-static single and cyclic indentation tests, such as the evolution of the dislocation microstructure and the deformation bands, are all included in hydrostatic pressure zone and plastic deformation zone under the indenter calculated in section of *Deformation behavior evolution*.

## Conclusion

To investigate cyclic elastoplastic deformation behaviors of austenite phase and ferrite phase underneath the indents, load-controlled cyclic indentation tests with Berkovich indenter were conducted. Based on the analysis of expansion model of spherical cavity, the relationship between the penetration depth and the distribution of deformation zones under the indenter was discussed. Besides, the deformation mechanisms in both phases under the indenter were provided by TEM observation. According to the experimental and analytical results, the main conclusions are concluded as follows:

1. With cycle number increasing, cyclic indentation tests on both austenite phase and ferrite phase experienced the transient state and quasi-steady state. The values and the increasing rates of the maximum penetration depth per cycle of ferrite phase were lower than those of austenite phase, suggesting ferrite phase had higher cyclic penetration resistance.
2. In cyclic indentation tests on both austenite phase and ferrite phase, the total absorbed energy contained two aspects: one is the dissipated plastic energy, promoting the propagation of elastoplastic deformation under the indenter during the loading phase, and having the same trend of the increment value of the maximum penetration depth per cycle. The other was the elastic recovered energy, releasing during the unloading phase, and having the same trend of the amplitude value of the penetration depth per cycle.
3. After the quasi-static single load, dislocations nucleated and concentrated under the indenter in both austenite phase and ferrite phase. After 300 cycles loading-unloading processes, no significant generation and concentration of dislocation were observed under the indenter and the dislocations propagated to the further region in both phases. This explained why the increment value and the dissipated plastic energy per cycle were much greater at the first few cycles while those were much lower at the quasi-steady state. Besides, slip bands generated and propagated in ferrite phase after single and cyclic indentation load, respectively. All deformation behaviors occurred in hydrostatic pressure zone and plastic deformation zone under the indenter.

## Electronic supplementary material

Supplementary information

## References

[1] Terao H (1986). Structure and mechanical properties of high-manganese dual-phase steels. Journal of Materials Science.

[2] Nelson DE, Baeslack Iii WA, Lippold JC (1997). Characterization of the Weld Structure in a Duplex Stainless Steel Using Color Metallography. Materials Characterization.

[3] Ravindranath K, Malhotra SN (1995). The influence of aging on the intergranular corrosion of 22 chromium-5 nickel duplex stainless steel. Corrosion Science.

[4] Huang Q (2016). On the dynamic mechanical property and deformation mechanism of as-extruded Mg-Sn-Ca alloys under tension. Materials Science and Engineering: A.

[5] Stölken JS, Evans AG (1998). A microbend test method for measuring the plasticity length scale. Acta Materialia.

[6] Yilmazer H (2013). Mechanical properties of a medical beta-type titanium alloy with specific microstructural evolution through high-pressure torsion. Materials science & engineering. C, Materials for biological applications.

[7] Oliver WC, Pharr GM (2011). Measurement of hardness and elastic modulus by instrumented indentation: Advances in understanding and refinements to methodology. Journal of Materials Research.

[8] Oliver WC, Pharr GM (2011). An improved technique for determining hardness and elastic modulus using load and displacement sensing indentation experiments. Journal of Materials Research.

[9] Yan FK, Zhang BB, Wang HT, Tao NR, Lu K (2016). Nanoindentation characterization of nano-twinned grains in an austenitic stainless steel. Scripta Materialia.

[10] Csanádi T, Bl’anda M, Chinh NQ, Hvizdoš P, Dusza J (2015). Orientation-dependent hardness and nanoindentation-induced deformation mechanisms of WC crystals. Acta Materialia.

[11] Guo LQ, Lin MC, Qiao LJ, Volinsky AA (2013). Ferrite and austenite phase identification in duplex stainless steel using SPM techniques. Applied Surface Science.

[12] Misra RDK, Zhang Z, Jia Z, Somani MC, Karjalainen LP (2010). Probing deformation processes in near-defect free volume in high strength–high ductility nanograined/ultrafine-grained (NG/UFG) metastable austenitic stainless steels. Scripta Materialia.

[13] Jia Y, Xuan FZ, Yang F (2015). Viscoplastic response of tooth enamel under cyclic microindentation. Materials science & engineering. C, Materials for biological applications.

[14] Schwarm SC, Kolli RP, Aydogan E, Mburu S, Ankem S (2017). Characterization of phase properties and deformation in ferritic-austenitic duplex stainless steels by nanoindentation and finite element method. Materials Science and Engineering: A.

[15] Guo E-Y (2014). Mechanical characterization of microconstituents in a cast duplex stainless steel by micropillar compression. Materials Science and Engineering: A.

[16] Chen J (2016). Effects of loading rate on development of pile-up during indentation creep of polycrystalline copper. Materials Science and Engineering: A.

[17] Xie KY (2011). Insight into the deformation mechanisms of α-Fe at the nanoscale. Scripta Materialia.

[18] Xie KY (2013). The effect of pre-existing defects on the strength and deformation behavior of α-Fe nanopillars. Acta Materialia.

[19] Misra RDK (2011). Nanomechanical insights into the deformation behavior of austenitic alloys with different stacking fault energies and austenitic stability. Materials Science and Engineering: A.

[20] Yang F, Peng L, Okazaki K (2007). Cyclic indentation in aluminum. Journal of Materials Science.

[21] Amini, A., Cheng, C., Kan, Q., Naebe, M. & Song, H. Phase Transformation Evolution in NiTi Shape Memory Alloy under Cyclic Nanoindentation Loadings at Dissimilar Rates. *Scientific Reports* **3**, 10.1038/srep03412 (2013). 10.1038/srep03412 PMC3861797 24336228

[22] Jia Y, Xuan F-z, Chen X, Yang F (2014). Finite element analysis of the cyclic indentation of bilayer enamel. Journal of Physics D: Applied Physics.

[23] (ASME E562-11, 2008).

[24] Johnson, K. L. *Contact Mechanics*. (Cambridge University, 1985).

[25] Hill, R. *The Mathematical Theory of Plasticity*. (Clarendon Press, 1950).

[26] Ahn TH (2010). Investigation of strain-induced martensitic transformation in metastable austenite using nanoindentation. Scripta Materialia.

[27] He BB (2013). Nanoindentation investigation on the mechanical stability of individual austenite grains in a medium-Mn transformation-induced plasticity steel. Scripta Materialia.

[28] Qiao X, Han L, Zhang W, Gu J (2015). Nano-indentation investigation on the mechanical stability of individual austenite in high-carbon steel. Materials Characterization.

[29] Kim Y, Ahn T-H, Suh D-W, Han HN (2015). Variant selection during mechanically induced martensitic transformation of metastable austenite by nanoindentation. Scripta Materialia.

[30] Roa JJ, Fargas G, Mateo A, Jiménez-Piqué E (2015). Dependence of nanoindentation hardness with crystallographic orientation of austenite grains in metastable stainless steels. Materials Science and Engineering: A.

[31] Han HN, Lee CG, Oh C-S, Lee T-H, Kim S-J (2004). A model for deformation behavior and mechanically induced martensitic transformation of metastable austenitic steel. Acta Materialia.

[32] Dumay A, Chateau JP, Allain S, Migot S, Bouaziz O (2008). Influence of addition elements on the stacking-fault energy and mechanical properties of an austenitic Fe–Mn–C steel. Materials Science and Engineering: A.

[33] Kim J-K, De Cooman BC (2016). Stacking fault energy and deformation mechanisms in Fe-xMn-0.6C-yAl TWIP steel. Materials Science and Engineering: A.

[34] Pierce DT, Jiménez JA, Bentley J, Raabe D, Wittig JE (2015). The influence of stacking fault energy on the microstructural and strain-hardening evolution of Fe–Mn–Al–Si steels during tensile deformation. Acta Materialia.

[35] Dai Q-X, Wang An-Dong CX-N, Luo X-M (2002). Stacking fault energy of cryogenic austenitic steels. Chinese Physics.

[36] Choi JY (2016). Effects of the strain rate on the tensile properties of a TRIP-aided duplex stainless steel. Materials Science and Engineering: A.

[37] Fisher JC, Hart EW, Pry RH (1952). Theory of Slip-Band Formation. Physical Review.

