# Supplementary material for: Micro-deformation evolutions of the constituent phases in duplex stainless steel during cyclic nanoindentation
Source: Sci Rep. 2018 Apr 18;8:6199. doi: 10.1038/s41598-018-24589-4 (PMC5906455; doi:10.1038/s41598-018-24589-4)
Supplement: Supplementary file 1 — Supplementary information [file 41598_2018_24589_MOESM1_ESM.docx]

**Supplementary information**

**Micro-deformation evolutions of the constituent phases in duplex stainless steel during cyclic nanoindentation**

Yuan-Yuan Cui, Yun-Fei Jia*, Fu-Zhen Xuan*

Key Laboratory of Pressure System and Safety, MOE, School of Mechanical and Power Engineering, East China University of Science and Technology, Shanghai 200237, P.R. China

*Corresponding author: *E-mail address*: [yfjia@ecust.edu.cn](mailto:yfjia@ecust.edu.cn) (Yun-Fei Jia)

fzxuan@ecust.edu.cn (Fu-Zhen Xuan)

**Supplementary Materials and Methods**

| Phase | C | O | Si | Cr | Mn | Ni | Mo | Fe |
| --- | --- | --- | --- | --- | --- | --- | --- | --- |
| Austenite | 0.56 | 1.26 | 0.42 | 25.50 | 0.80 | 6.13 | 3.64 | Bal. |
| Ferrite | 0.55 | 0.86 | 0.38 | 25.19 | 0.80 | 7.89 | 2.87 | Bal. |

Table S1. The chemical compositions of the respective phases in the studied duplex stainless steel.

Table S1 lists the chemical compositions of the respective phases in as-received duplex stainless steel from Baosteel Stainless Steel Co., Ltd.


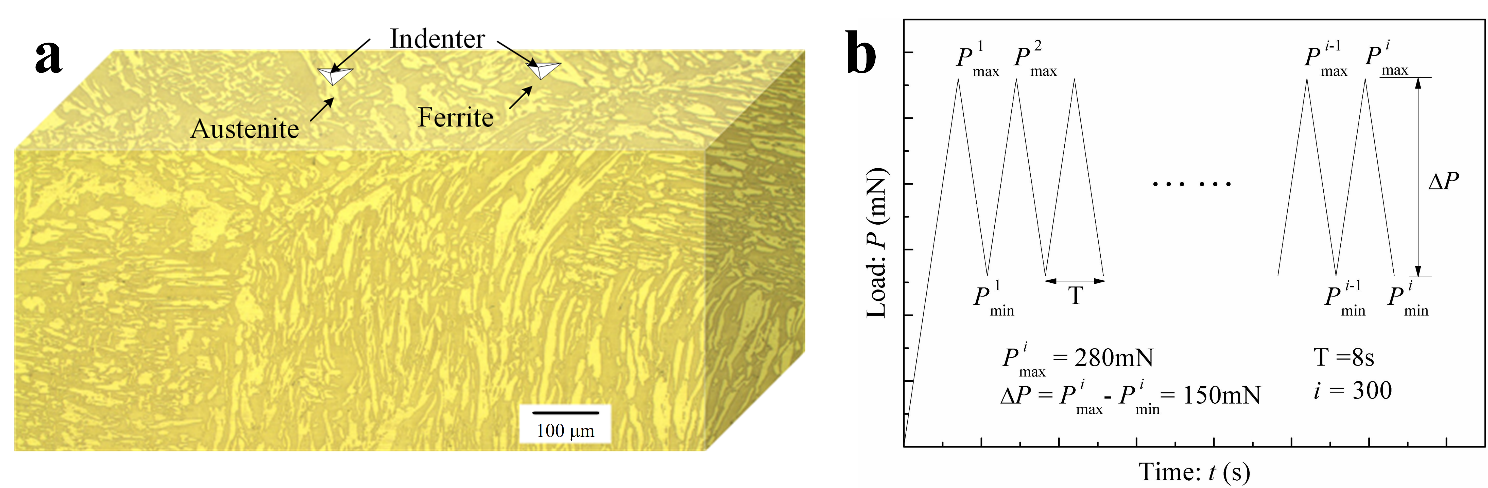


Figure S1. (a) Schematic of the three-dimensional microstructure of studied stainless steel observed by the optional microscope and indenting positions on the respective phases, and (b) schematic of the cyclic nanoindentation loading.

Figure S1 (a) shows three-dimensional microstructure of studied stainless steel observed by the optional microscope. The loading positions were pointed out in Fig. S1(a). The loading/unloading path followed triangular wave as shown in Figure S2. (b). The cyclic indentation tests were performed in load-controlled mode.


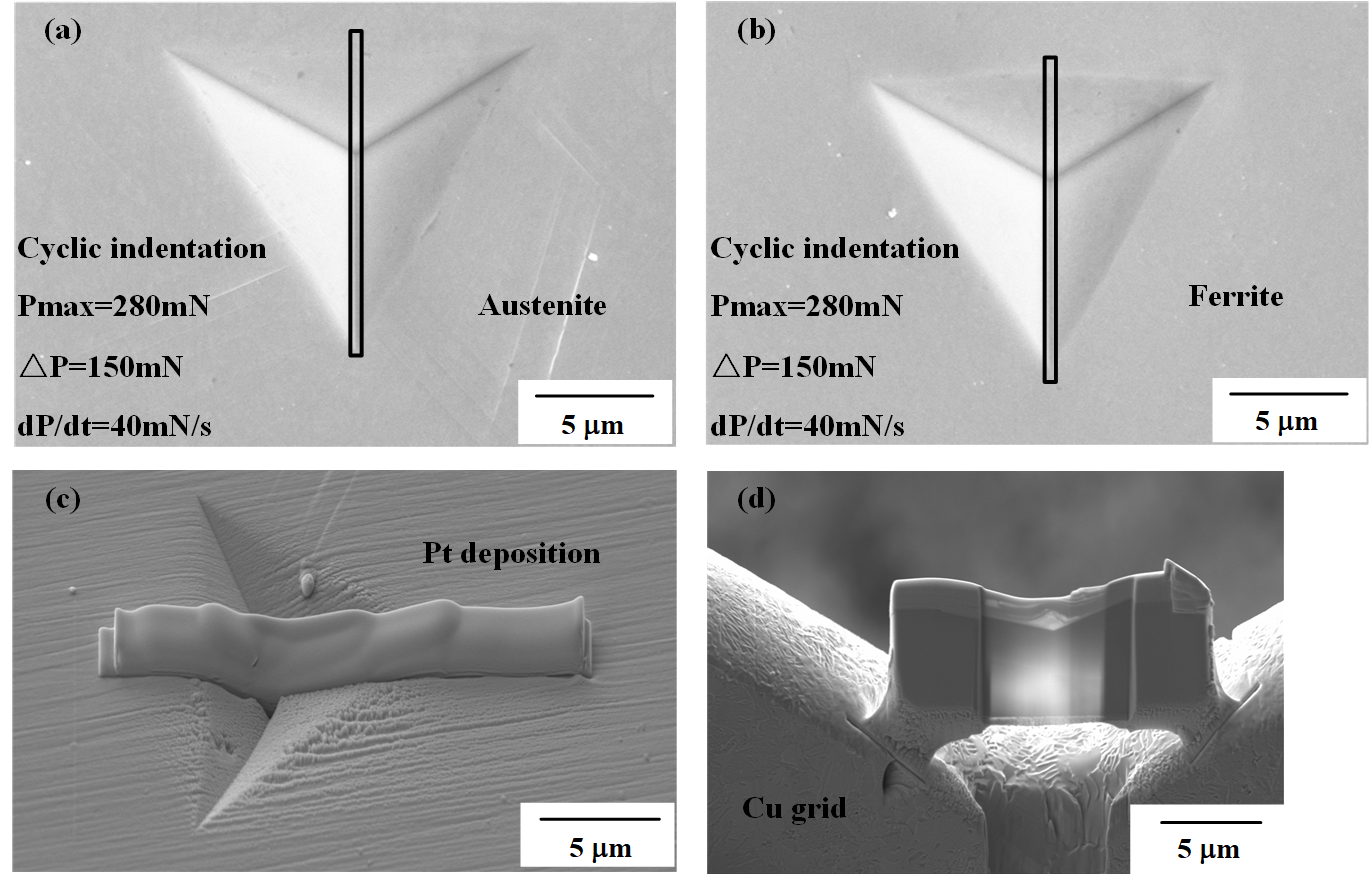


Figure S2. Representative SEM images of indents after 300 cycles of nanoindentation and the FIB milling directions of the cross sectional TEM thin foils on (a) austenite phase and (b) ferrite phase. (c) Protective platinum layer deposited on the indent surface. (d) Thin foil on the Cu grid prepared for TEM observation.

Figure S2 shows the location and process of FIB milling. The protective platinum layer deposited on the indent surface to protect the specimen surface from the ion beam damage. The cross sectional TEM thin foils on both austenite phase and ferrite phase were milled beneath the indents after the action of quasi-static load with *P_max_* of 280mN and cyclic load with *P_max_* of 280mN and $\Delta P$ of 150mN. Moreover, Fig.S2 (a) and (b) show representative SEM micrographs of indentations on austenite phase and ferrite phase after 300 cycles. No cracking around the impressions was observed, suggesting only the elasto-plastic deformation under the indenter occurred during the tests. When the solid indentation contains large plastic deformation, resulting from its plastic flows along the faces of the indenter, the major contact-induced deformations must overcome the mechanical constraints of the free surface of the solid and result in the contact profile of piling-up around the impression, as shown in the inserts of Fig.1(b). Piling-up is caused by the plastic flow of materials out of the indented region and thus its formation and development are related to the plastic deformation mechanisms[^1^](#_ENREF_1). The effect of the change of contact area due to pile-up on the measurement of elastic modulus and hardness is neglectable in this study.

| Parameters |  | $\Delta P$- controlled tests | | | | |  | $P_{max}$-controlled tests | | | | |
| --- | --- | --- | --- | --- | --- | --- | --- | --- | --- | --- | --- | --- |
| $P_{max}$(mN) |  | 160 | 200 | 240 | 280 | 320 |  | 200 | 200 | 200 | 200 | 200 |
| $\Delta P$(mN) |  | 150 | 150 | 150 | 150 | 150 |  | 30 | 70 | 110 | 150 | 190 |

Table S2. All loading conditions of cyclic indentation tests with constant ${dP}/{dt}$ of 40mN/s on austenite phase and ferrite phase.

Table S2 lists the entire loading conditions of cyclic indentation tests. The effects of loading parameters of $P_{max}$ and $\Delta P$ on the cyclic deformation behaviors of austenite phase and ferrite phase are studied. The $P_{max}$ was in the range of 160mN to 320mN with $\Delta P$ of 150mN, and the $\Delta P$ was in the range of 30mN to 190mN with $P_{max}$of 200mN. The loading/unloading rates for all tests are 40mN/s.

**Supplementary Discussions**

**Distribution of deformation zone**


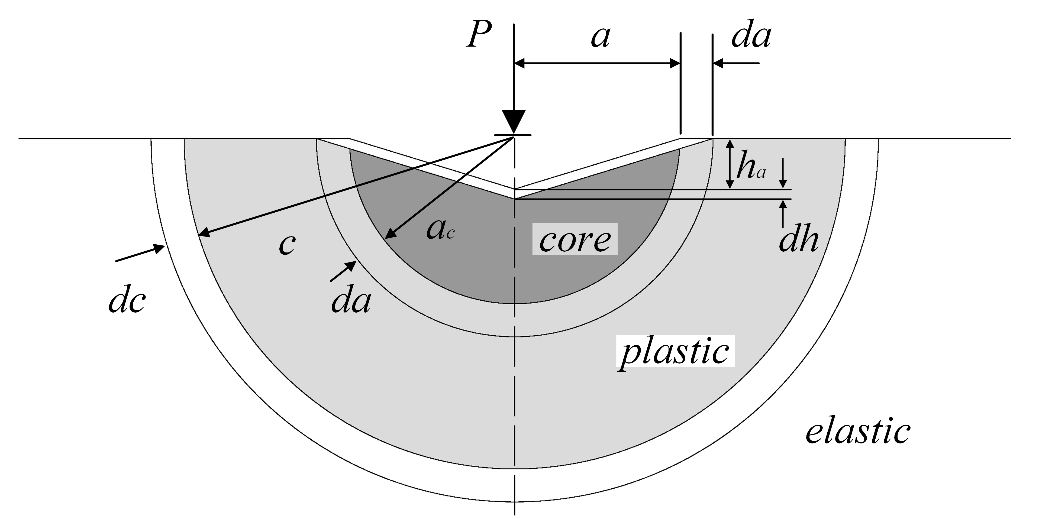


Figure S3. Expanding cavity model schematic.[^2^](#_ENREF_2)

Figure S3 shows the schematic of deformation zone by spherical cavity model in an elastic-plastic material[^2^](#_ENREF_2). The distribution of deformation zone is depicted by the hemispherical radial mode. The hemispherical core of material directly beneath the indenter with radius equal to the contact circle, *a_c_*, is incompressible. Outside the hydrostatic pressure zone, a hemispherical plastic zone of radius, *c*, is the elastic-plastic deformation zone, which is boundary of the plastic-elastic deformation zone as well.

**Effects of the maximum indentation load**

(b)

(a)







Figure S4. $h_{max}^{i}$ as a function of *i* for the cyclic indentation of (a) austenite phase and (b) ferrite phase with different $P_{max}$ ($\Delta P$ of 150mN and ${dP}/{dt}$ of 40mN/s).

Figure S4 (a) and (b) show the variation of $h_{max}^{i}$ with *i* during the cyclic indentation tests with different $P_{max}$ on austenite phase and ferrite phase, respectively. The values of $h_{max}^{i}$ for the cyclic indentation tests on both austenite phase and ferrite phase increase with the cycle number, and the increasing rate of the maximum penetration depth, ${dh_{max}^{i}}/{dt}$, decreases with the cycle number. The increasing rates of $h_{max}^{i}$ for all cyclic indentation tests during 1~10 cycles, 10~30 cycles, 30~100 cycles, 100~200 cycles and 200~300 cycles, respectively, on respective phases are presented in Fig. S4. The increasing rates of austenite phase decrease from 4.620±0.698nm/s at the first 10 cycles to 0.157±0.030 nm/s at the last 100 cycles for different $P_{max}$, while those of ferrite phase decrease from 2.954±0.193 nm/s to 0.135±0.017 nm/s. Due to the higher cyclic penetration resistance of ferrite phase, the increasing rates of the maximum penetration depth of ferrite phase are lower than those of austenite phase at the same cycle.


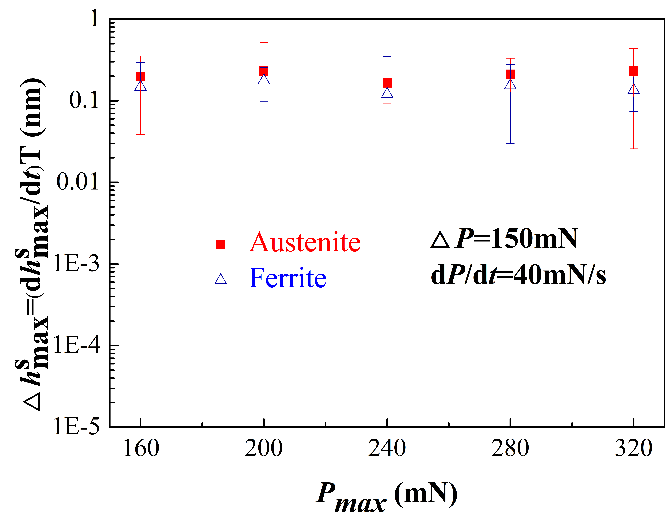


Figure S5. Dependence of ${\Delta h}_{max}^{s}$ on $P_{max}$ ($\Delta P$= 150mN and ${dP}/{dt}$ = 40mN/s) for austenite phase and ferrite phase.

Figure S5 shows the variation of the increasing value of the maximum penetration depth at the quasi-steady state, ${\Delta h}_{max}^{s}$, with the maximum indentation load $P_{max}$ for the cyclic indentation of austenite phase and ferrite phase with $\Delta P$= 150mN and ${dP}/{dt}$= 40mN/s. The value of ${\Delta h}_{max}^{s}$ is independent of $P_{max}$ under the experimental conditions for both austenite phase and ferrite phase, and the cyclic indentation of austenite phase produces larger penetration depths than the cyclic indentation of ferrite phase under the same cyclic loading at the quasi-steady state.


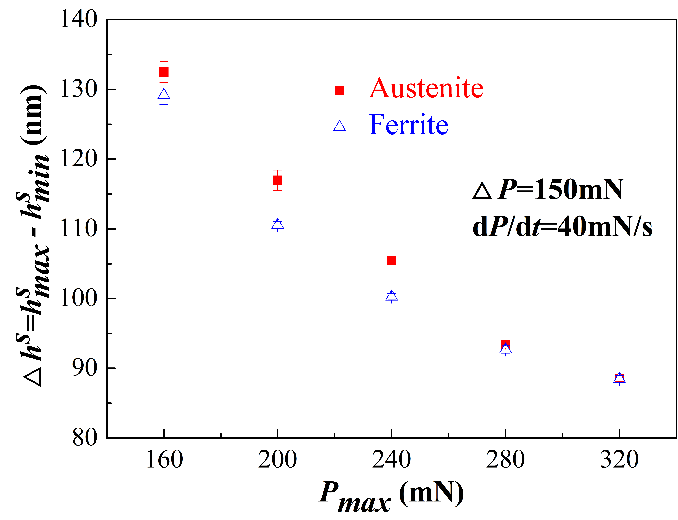


Figure S6. Dependence of ${\Delta h}^{s}$ on $P_{max}$($\Delta P$= 150mN and ${dP}/{dt}$= 40mN/s) for austenite phase and ferrite phase.

Figure S6 shows the dependence of the amplitude of the penetration depth at the quasi-steady state, ${\Delta h}_{max}^{s}$, on $P_{max}$ at $\Delta P$= 150mN and ${dP}/{dt}$= 40mN/s for the cyclic indentation on austenite phase and ferrite phase. The value of ${\Delta h}_{max}^{s}$ decreases with increasing the value of $P_{max}$. Note that, at the same $P_{max}$ and $\Delta P$, larger amplitude of penetration depth is produced on austenite phase than on ferrite phase at the quasi-steady state. This result suggests that there exists larger elastic recovery of austenite phase than that of ferrite phase under the same loading condition, which could be related to larger elastic modulus of austenite phase than that of ferrite phase.





Figure S7. Dependence of $E_{p}^{s}$ on $P_{max}$($\Delta P$= 150mN and ${dP}/{dt}$= 40mN/s) for austenite phase and ferrite phase.

Figure S7 shows the dependence of the plastic dissipated energy at the quasi-steady state, $E_{p}^{s}$, on $P_{max}$ for the cyclic indentation of austenite phase and ferrite phase. The value of $E_{p}^{s}$ decreases with increasing of the value of $P_{max}$. The values of $E_{p}^{s}$ of austenite phase are larger than those of ferrite phase at the same value of the maximum indentation load. The percentage difference of $E_{p}^{s}$ between the austenite phase and ferrite phase decreases with increasing the maximum load. Generally, austenite phase has greater ability to absorb plastic energy at the quasi-steady state during the cyclic indentation.





Figure S8. Dependence of $E_{e}^{s}$ on $P_{max}$($\Delta P$= 150mN and ${dP}/{dt}$= 40mN/s) for austenite phase and ferrite phase.

Figure S8 shows the dependence of the elastic recovery energy at the quasi-steady state, $E_{e}^{s}$, on $P_{max}$ for the cyclic indentation of austenite phase and ferrite phase. Contrary to the variation of ${\Delta h}^{s}$, the values of $E_{e}^{s}$ of both austenite phase and ferrite phase increase with $P_{max}$. With increasing the maximum load, the volume of indent increase. Thus, although the elastic recovery energy increases with the maximum load, the elastic recovery depth decreases. Under the action of same cyclic loading, at the quasi-steady state, austenite phase absorbed more elastic energy than ferrite phase, and produced larger elastic deformation.

**Effects of amplitude of the indentation load**


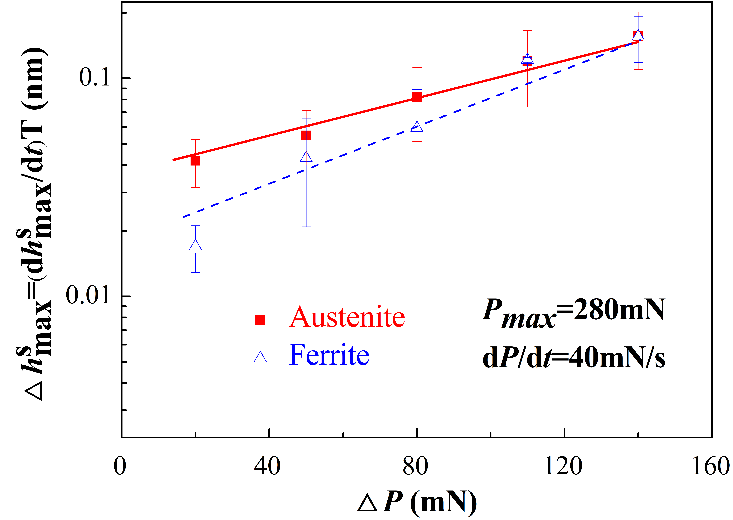


Fig S9. Variation of ${\Delta h}_{max}^{s}$ with $\Delta P$ for the cyclic indentation of the austenite phase and ferrite phase.

Figure S9 shows the dependence of the increasing value per cycle of the maximum penetration depth at the quasi-steady state, ${\Delta h}_{max}^{s}$, on the amplitude of the indentation load, $\Delta P$, during the cyclic indentation tests with $P_{max}$ of 200mN and ${dP}/{dt}$ of 40mN/s. The values of ${\Delta h}_{max}^{s}$ of both austenite phase and ferrite phase increase with the increase of $\Delta P$. The cyclic indentation of the austenite phase produces larger penetration depth per cycle than the cyclic indentation of the ferrite phase under the same load condition. The ferrite phase has larger resistance to the propagation of the plastic zone than the austenite phase under the same loading.


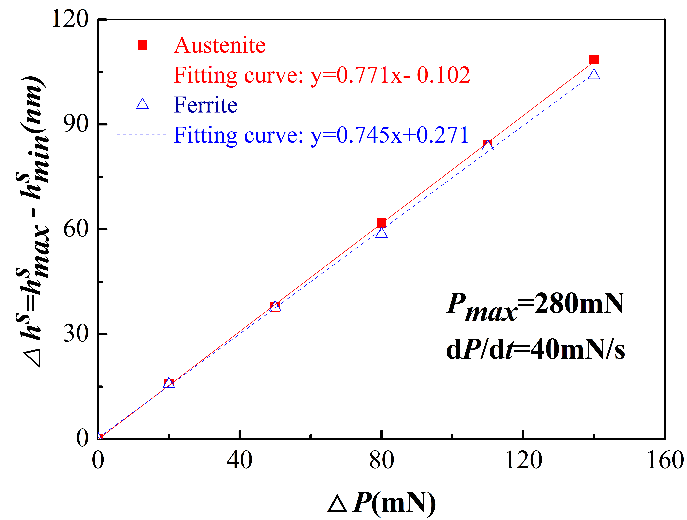


Fig S10. Variation of ${\Delta h}^{s}$ with $\Delta P$ for the cyclic indentation of the austenite phase and ferrite phase.

Figure S10 shows the dependence of the amplitude of the penetration depth at the quasi-steady state, ${\Delta h}^{s}$, on the amplitude of the cyclic indentation load, $\Delta P$, at $P_{max}$ =200 mN for the indentation on austenite phase and ferrite phase. The value of ${\Delta h}^{s}$ of both phases increases linearly with the increase of $\Delta P$, as expected. Austenite phase has a slightly larger elastic recovery depth due to its lower elastic modulus.


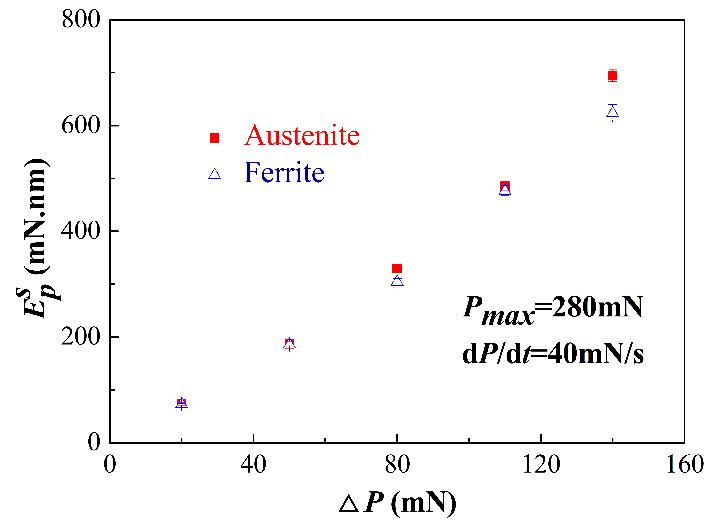


Fig S11. The plastic energy dissipation at the quasi-steady state of $E_{p}^{s}$ as a function of $\Delta P$ under $P_{max}$ =200 mN for the cyclic indentation of the austenite phase and ferrite phase.

Figure S11 shows the dependence of $E_{p}^{s}$ on $\Delta P.$ Under the action of cyclic loading with constant $P_{max}$ and ${dP}/{dt}$, the values of $E_{p}^{s}$ of both phases increase with the value of $\Delta P$. With increasing the value of $\Delta P$, the difference of the plastic energy dissipation between the two phases increases.


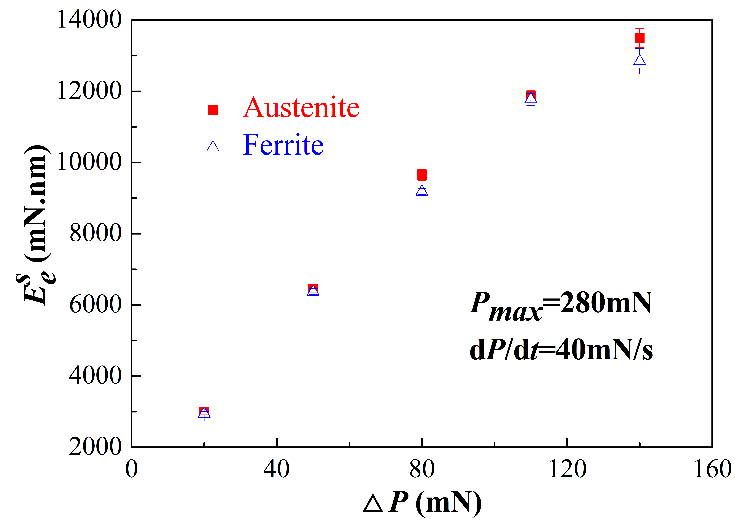


Fig S12. The elastic recovery energy at the quasi-steady state of $E_{e}^{s}$ as a function of $\Delta P$ under $P_{max}$ =200 mN for the cyclic indentation of the austenite phase and ferrite phase.

Figure S12 shows the dependence of $E_{e}^{s}$ on $\Delta P.$ The value of $E_{e}^{s}$ has same trend of ${\Delta h}^{s}$ shown in Fig. S8, which manifests that higher elastic recovery energy produce higher elastic recovery depth.

Reference

1 Chen, J. *et al.* Effects of loading rate on development of pile-up during indentation creep of polycrystalline copper. *Materials Science and Engineering: A* **656**, 216-221, doi:10.1016/j.msea.2016.01.042 (2016).

2 Johnson, K. L. *Contact Mechanics*. (Cambridge University, 1985).
